# 4-Chloro-*N*-(2,4-dimethyl­phen­yl)-2-methyl­benzene­sulfonamide

**DOI:** 10.1107/S1600536811036956

**Published:** 2011-09-17

**Authors:** Vinola Z. Rodrigues, Sabine Foro, B. Thimme Gowda

**Affiliations:** a Department of Chemistry, Mangalore University, Mangalagangotri 574 199, Mangalore, India; b Institute of Materials Science, Darmstadt University of Technology, Petersenstrasse 23, D-64287 Darmstadt, Germany

## Abstract

In the title compound, C_15_H_16_ClNO_2_S, the C—SO_2_—NH—C torsion angle is 67.45 (17)°. The two aromatic rings are tilted relative to each other by 44.5 (1)°. The crystal structure features inversion-related dimers linked by pairs of N—H⋯O hydrogen bonds..

## Related literature

For the preparation of the title compound, see: Savitha & Gowda (2006 ▶). For hydrogen-bonding modes of sulfonamides, see: Adsmond & Grant (2001 ▶). For studies of the effects of substituents on the structures and other aspects of *N*-(ar­yl)-amides, see: Arjunan *et al.* (2004 ▶); Gowda *et al.* (2000 ▶), on *N*-(ar­yl)-methane­sulfonamides, see: Gowda *et al.* (2007 ▶) and on *N*-(ar­yl)-aryl­sulfonamides, see: Gelbrich *et al.* (2007 ▶); Gowda *et al.* (2010 ▶); Perlovich *et al.* (2006 ▶); Rodrigues *et al.* (2011 ▶).
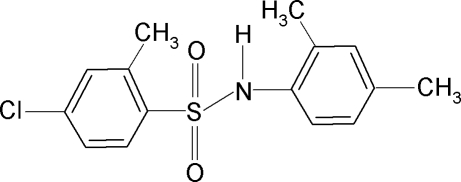


## Experimental

### 

#### Crystal data

C_15_H_16_ClNO_2_S
*M* *_r_* = 309.80
Monoclinic, 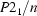

*a* = 8.2578 (7) Å
*b* = 12.665 (1) Å
*c* = 14.299 (2) Å
β = 92.187 (7)°
*V* = 1494.4 (3) Å^3^
*Z* = 4
Mo *K*α radiation
μ = 0.40 mm^−1^
*T* = 293 K
0.48 × 0.30 × 0.20 mm

#### Data collection

Oxford Diffraction Xcalibur diffractometer with a Sapphire CCD detector
Absorption correction: multi-scan (*CrysAlis RED*; Oxford Diffraction, 2009 ▶) *T*_min_ = 0.833, *T*_max_ = 0.925
5555 measured reflections
3041 independent reflections
2307 reflections with *I* > 2σ(*I*)
*R*_int_ = 0.014

#### Refinement

*R*[*F* ^2^ > 2σ(*F* ^2^)] = 0.040
*wR*(*F* ^2^) = 0.114
*S* = 1.06
3041 reflections
187 parameters
1 restraint
H atoms treated by a mixture of independent and constrained refinement
Δρ_max_ = 0.28 e Å^−3^
Δρ_min_ = −0.45 e Å^−3^

### 

Data collection: *CrysAlis CCD* (Oxford Diffraction, 2009 ▶); cell refinement: *CrysAlis RED* (Oxford Diffraction, 2009 ▶); data reduction: *CrysAlis RED*; program(s) used to solve structure: *SHELXS97* (Sheldrick, 2008 ▶); program(s) used to refine structure: *SHELXL97* (Sheldrick, 2008 ▶); molecular graphics: *PLATON* (Spek, 2009 ▶); software used to prepare material for publication: *SHELXL97*.

## Supplementary Material

Crystal structure: contains datablock(s) I, global. DOI: 10.1107/S1600536811036956/bt5641sup1.cif

Structure factors: contains datablock(s) I. DOI: 10.1107/S1600536811036956/bt5641Isup2.hkl

Supplementary material file. DOI: 10.1107/S1600536811036956/bt5641Isup3.cml

Additional supplementary materials:  crystallographic information; 3D view; checkCIF report

## Figures and Tables

**Table 1:** Hydrogen-bond geometry (Å, °)

*D*—H⋯*A*	*D*—H	H⋯*A*	*D*⋯*A*	*D*—H⋯*A*
N1—H1*N*⋯O2^i^	0.83 (2)	2.27 (2)	3.072 (2)	162 (2)

Symmetry code: (i) 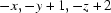.

**Fig. 1.**
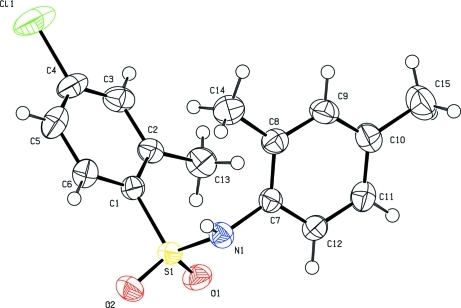
Molecular structure of the title compound, showing the atom labelling scheme and displacement ellipsoids are drawn at the 50% probability level.

**Fig. 2.**
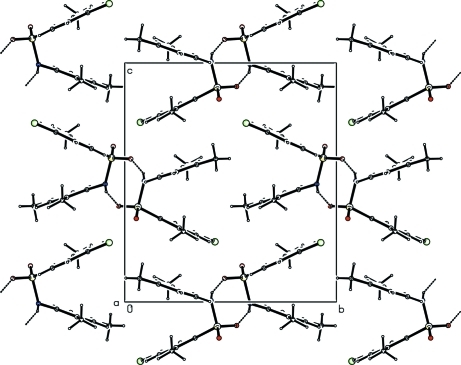
Molecular packing of the title compound with hydrogen bonding shown as dashed lines.

## supplementary crystallographic information

### Comment

The sulfonamide moieties are the constituents of many biologically significant compounds. The hydrogen bonding preferences of sulfonamides have been investigated (Adsmond & Grant, 2001). As part of our studies on the substituent effects on the structures and other aspects of *N*-(aryl)-amides (Arjunan *et al.*, 2004; Gowda *et al.*, 2000), *N*-(aryl)-methanesulfonamides (Gowda *et al.*, 2007) and *N*-(aryl)-arylsulfonamides (Gowda *et al.*, 2010; Rodrigues *et al.*, 2011), in the present work, the crystal structure of 4-Chloro-2-methyl-*N*-(2,4-dimethylphenyl)benzenesulfonamide (I) has been determined (Fig. 1).

In (I), the N—H bond in the C—SO_2_—NH—C segment is *syn* with respect to the *ortho*-methyl group in the anilino benzene ring and orients towards the *ortho*-methyl group in the sulfonyl benzene ring. Further, the sulfonyl group orients itself away from the *ortho*- methyl groups in both the rings. The molecule is bent at the S atom with the C—SO_2_—NH—C torsion angle of 67.5 (2), compared to the values of -66.8 (3)° and 70.3 (3)° in the two independent molecules of 4-Chloro-2-methyl-*N*-(2,3-dimethylphenyl)benzenesulfonamide (II) (Rodrigues *et al.*, 2011), and -61.9 (4)° and 69.7 (4)° in the two molecules of 4-chloro-2-methyl-*N*-(phenyl)-benzenesulfonamide (III) and -76.5 (5)° and -48.3 (4)° in 4-chloro-2-methyl-*N*-(4-methylphenyl)-benzenesulfonamide (IV) (Gowda *et al.*, 2010).

The sulfonyl and the aniline benzene rings are tilted relative to each other by 44.5 (1)°, compared to the values of 44.1 (1)° in molecule 1 and 39.7 (1)° in molecule 2 of (II), 86.6 (2)° and 83.0 (2)° in the two independent molecules of (III), and 76.6 (2)° in molecule 1 and 70.7 (2)° in molecule 2 of (IV).

The other bond parameters in (I) are similar to those observed in (II), (III) and other aryl sulfonamides (Perlovich *et al.*, 2006; Gelbrich *et al.*, 2007).

In the crystal, the intermolecular N–H···O hydrogen bonds (Table 1) link the molecules into dimeric chains. Part of the crystal structure is shown in Fig. 2.

### Experimental

The solution of *m*-chlorotoluene (10 ml) in chloroform (40 ml) was treated dropwise with chlorosulfonic acid (25 ml) at 0 ° C. After the initial evolution of hydrogen chloride subsided, the reaction mixture was brought to room temperature and poured into crushed ice in a beaker. The chloroform layer was separated, washed with cold water and allowed to evaporate slowly. The residual 2-methyl-4-chlorobenzenesulfonylchloride was treated with 2,4-dimethylaniline in the stoichiometric ratio and boiled for ten minutes. The reaction mixture was then cooled to room temperature and added to ice cold water (100 ml). The resultant solid 4-chloro-2-methyl-*N*- (2,4-dimethylphenyl)-benzenesulfonamide was filtered under suction and washed thoroughly with cold water. It was then recrystallized to constant melting point from dilute ethanol. The purity of the compound was checked and characterized by recording its infrared and NMR spectra (Savitha & Gowda, 2006).

Rod like light pink single crystals used in X-ray diffraction studies were grown in ethanolic solution by slow evaporation at room temperature.

### Refinement

The H atom  of the NH group  was located in a difference map and refined with the N—H  distance restrained to  0.86 (2) %A and *U*_iso_(H)= 1.2*U*_eq_(N). The other H atoms were positioned with idealized geometry using a riding model with   aromatic C—H = 0.93Å and methyl C—H = 0.96 Å. Their displacement parameters were set to 1.2*U*_eq_(C_aromatic_) or 1.5*U*_eq_(C_methyl_).

### Crystal data

**Table:**

C_15_H_16_ClNO_2_S	*F*(000) = 648
*M**_r_* = 309.80	*D*_x_ = 1.377 Mg m^−^^3^
Monoclinic, *P*2_1_/*n*	Mo *K*α radiation, λ = 0.71073 Å
Hall symbol: -P 2yn	Cell parameters from 1699 reflections
*a* = 8.2578 (7) Å	θ = 2.8–27.7°
*b* = 12.665 (1) Å	µ = 0.40 mm^−^^1^
*c* = 14.299 (2) Å	*T* = 293 K
β = 92.187 (7)°	Rod, light pink
*V* = 1494.4 (3) Å^3^	0.48 × 0.30 × 0.20 mm
*Z* = 4	

### Data collection

**Table:**

Oxford Diffraction Xcalibur diffractometer with a Sapphire CCD detector	3041 independent reflections
Radiation source: fine-focus sealed tube	2307 reflections with *I* > 2σ(*I*)
graphite	*R*_int_ = 0.014
Rotation method data acquisition using ω scans	θ_max_ = 26.3°, θ_min_ = 2.8°
Absorption correction: multi-scan (*CrysAlis RED*; Oxford Diffraction, 2009)	*h* = −10→8
*T*_min_ = 0.833, *T*_max_ = 0.925	*k* = −8→15
5555 measured reflections	*l* = −16→17

### Refinement

**Table:**

Refinement on *F*^2^	Primary atom site location: structure-invariant direct methods
Least-squares matrix: full	Secondary atom site location: difference Fourier map
*R*[*F*^2^ > 2σ(*F*^2^)] = 0.040	Hydrogen site location: inferred from neighbouring sites
*wR*(*F*^2^) = 0.114	H atoms treated by a mixture of independent and constrained refinement
*S* = 1.06	*w* = 1/[σ^2^(*F*_o_^2^) + (0.0574*P*)^2^ + 0.4618*P*] where *P* = (*F*_o_^2^ + 2*F*_c_^2^)/3
3041 reflections	(Δ/σ)_max_ = 0.014
187 parameters	Δρ_max_ = 0.28 e Å^−^^3^
1 restraint	Δρ_min_ = −0.45 e Å^−^^3^

### Special details

**Table:**

Geometry. All e.s.d.'s (except the e.s.d. in the dihedral angle between two l.s. planes) are estimated using the full covariance matrix. The cell e.s.d.'s are taken into account individually in the estimation of e.s.d.'s in distances, angles and torsion angles; correlations between e.s.d.'s in cell parameters are only used when they are defined by crystal symmetry. An approximate (isotropic) treatment of cell e.s.d.'s is used for estimating e.s.d.'s involving l.s. planes.
Refinement. Refinement of *F*^2^ against ALL reflections. The weighted *R*-factor *wR* and goodness of fit *S* are based on *F*^2^, conventional *R*-factors *R* are based on *F*, with *F* set to zero for negative *F*^2^. The threshold expression of *F*^2^ > σ(*F*^2^) is used only for calculating *R*-factors(gt) *etc*. and is not relevant to the choice of reflections for refinement. *R*-factors based on *F*^2^ are statistically about twice as large as those based on *F*, and *R*- factors based on ALL data will be even larger.

### Fractional atomic coordinates and isotropic or equivalent isotropic displacement parameters (Å^2^)

**Table:**

	*x*	*y*	*z*	*U*_iso_*/*U*_eq_	
C1	0.0698 (2)	0.33223 (15)	0.84907 (13)	0.0316 (4)	
C2	0.1364 (2)	0.24190 (16)	0.80917 (14)	0.0342 (4)	
C3	0.0279 (3)	0.16309 (18)	0.77931 (15)	0.0437 (5)	
H3	0.0670	0.1019	0.7521	0.052*	
C4	−0.1362 (3)	0.17474 (19)	0.78958 (15)	0.0449 (6)	
C5	−0.2009 (3)	0.2637 (2)	0.82802 (15)	0.0472 (6)	
H5	−0.3123	0.2706	0.8335	0.057*	
C6	−0.0973 (2)	0.34208 (18)	0.85809 (14)	0.0395 (5)	
H6	−0.1388	0.4027	0.8849	0.047*	
C7	0.3384 (2)	0.32116 (16)	1.03000 (13)	0.0317 (4)	
C8	0.2795 (2)	0.22550 (17)	1.06210 (14)	0.0372 (5)	
C9	0.3915 (3)	0.14679 (18)	1.08376 (15)	0.0433 (5)	
H9	0.3538	0.0824	1.1053	0.052*	
C10	0.5574 (3)	0.15958 (18)	1.07479 (15)	0.0425 (5)	
C11	0.6109 (2)	0.25634 (19)	1.04392 (15)	0.0428 (5)	
H11	0.7213	0.2675	1.0378	0.051*	
C12	0.5036 (2)	0.33656 (17)	1.02208 (14)	0.0389 (5)	
H12	0.5420	0.4014	1.0019	0.047*	
C13	0.3156 (3)	0.22355 (19)	0.79533 (17)	0.0481 (6)	
H13A	0.3719	0.2195	0.8552	0.058*	
H13B	0.3585	0.2809	0.7601	0.058*	
H13C	0.3297	0.1586	0.7620	0.058*	
C14	0.1020 (3)	0.2057 (2)	1.07421 (19)	0.0561 (7)	
H14A	0.0466	0.2040	1.0140	0.067*	
H14B	0.0579	0.2612	1.1112	0.067*	
H14C	0.0881	0.1393	1.1052	0.067*	
C15	0.6731 (3)	0.0707 (2)	1.0985 (2)	0.0637 (7)	
H15A	0.6144	0.0054	1.1004	0.076*	
H15B	0.7255	0.0838	1.1585	0.076*	
H15C	0.7532	0.0665	1.0517	0.076*	
N1	0.2318 (2)	0.40758 (14)	1.00646 (12)	0.0354 (4)	
H1N	0.151 (2)	0.4122 (18)	1.0385 (14)	0.042*	
O1	0.33449 (17)	0.44561 (12)	0.84920 (11)	0.0441 (4)	
O2	0.08361 (18)	0.52847 (11)	0.90148 (11)	0.0455 (4)	
Cl1	−0.26595 (10)	0.07375 (6)	0.75182 (5)	0.0759 (3)	
S1	0.18711 (6)	0.43758 (4)	0.89731 (3)	0.03321 (16)	

### Atomic displacement parameters (Å^2^)

**Table:**

	*U*^11^	*U*^22^	*U*^33^	*U*^12^	*U*^13^	*U*^23^
C1	0.0336 (10)	0.0320 (11)	0.0290 (10)	−0.0024 (8)	−0.0015 (7)	0.0017 (8)
C2	0.0403 (11)	0.0317 (11)	0.0308 (10)	0.0007 (9)	0.0024 (8)	0.0002 (9)
C3	0.0619 (14)	0.0339 (12)	0.0354 (11)	−0.0034 (10)	0.0021 (9)	−0.0046 (10)
C4	0.0546 (13)	0.0478 (14)	0.0322 (11)	−0.0248 (11)	−0.0025 (9)	0.0013 (10)
C5	0.0353 (11)	0.0657 (16)	0.0406 (12)	−0.0108 (11)	−0.0009 (9)	−0.0015 (12)
C6	0.0356 (11)	0.0445 (13)	0.0383 (11)	0.0018 (9)	0.0001 (8)	−0.0044 (10)
C7	0.0355 (10)	0.0320 (11)	0.0272 (9)	0.0000 (8)	−0.0027 (7)	−0.0011 (8)
C8	0.0404 (11)	0.0386 (12)	0.0327 (11)	−0.0053 (9)	0.0018 (8)	0.0011 (9)
C9	0.0541 (13)	0.0342 (12)	0.0414 (12)	−0.0036 (10)	−0.0006 (9)	0.0063 (10)
C10	0.0494 (13)	0.0404 (13)	0.0374 (11)	0.0079 (10)	−0.0034 (9)	−0.0016 (10)
C11	0.0336 (10)	0.0524 (14)	0.0423 (12)	0.0002 (10)	−0.0011 (9)	0.0015 (11)
C12	0.0380 (11)	0.0384 (12)	0.0399 (11)	−0.0064 (9)	−0.0045 (8)	0.0034 (9)
C13	0.0464 (12)	0.0430 (13)	0.0553 (14)	0.0091 (10)	0.0082 (10)	−0.0100 (11)
C14	0.0448 (13)	0.0586 (16)	0.0652 (16)	−0.0102 (12)	0.0060 (11)	0.0170 (13)
C15	0.0659 (16)	0.0524 (17)	0.0719 (18)	0.0171 (13)	−0.0093 (13)	0.0004 (14)
N1	0.0373 (9)	0.0339 (10)	0.0347 (9)	0.0037 (7)	−0.0005 (7)	−0.0011 (8)
O1	0.0428 (8)	0.0440 (9)	0.0455 (9)	−0.0114 (7)	0.0024 (6)	0.0056 (7)
O2	0.0534 (9)	0.0288 (8)	0.0536 (10)	0.0061 (7)	−0.0073 (7)	0.0006 (7)
Cl1	0.0949 (5)	0.0740 (5)	0.0581 (4)	−0.0525 (4)	−0.0050 (4)	−0.0035 (4)
S1	0.0372 (3)	0.0270 (3)	0.0350 (3)	−0.0018 (2)	−0.00290 (19)	0.0013 (2)

### Geometric parameters (Å, °)

**Table:**

C1—C6	1.396 (3)	C10—C11	1.381 (3)
C1—C2	1.400 (3)	C10—C15	1.507 (3)
C1—S1	1.7731 (19)	C11—C12	1.376 (3)
C2—C3	1.397 (3)	C11—H11	0.9300
C2—C13	1.519 (3)	C12—H12	0.9300
C3—C4	1.377 (3)	C13—H13A	0.9600
C3—H3	0.9300	C13—H13B	0.9600
C4—C5	1.371 (3)	C13—H13C	0.9600
C4—Cl1	1.741 (2)	C14—H14A	0.9600
C5—C6	1.369 (3)	C14—H14B	0.9600
C5—H5	0.9300	C14—H14C	0.9600
C6—H6	0.9300	C15—H15A	0.9600
C7—C12	1.387 (3)	C15—H15B	0.9600
C7—C8	1.390 (3)	C15—H15C	0.9600
C7—N1	1.436 (2)	N1—S1	1.6349 (18)
C8—C9	1.387 (3)	N1—H1N	0.829 (15)
C8—C14	1.503 (3)	O1—S1	1.4243 (15)
C9—C10	1.390 (3)	O2—S1	1.4364 (15)
C9—H9	0.9300		
			
C6—C1—C2	121.03 (18)	C10—C11—H11	119.5
C6—C1—S1	115.10 (15)	C11—C12—C7	120.3 (2)
C2—C1—S1	123.77 (15)	C11—C12—H12	119.8
C3—C2—C1	116.80 (18)	C7—C12—H12	119.8
C3—C2—C13	117.88 (19)	C2—C13—H13A	109.5
C1—C2—C13	125.31 (18)	C2—C13—H13B	109.5
C4—C3—C2	120.9 (2)	H13A—C13—H13B	109.5
C4—C3—H3	119.6	C2—C13—H13C	109.5
C2—C3—H3	119.6	H13A—C13—H13C	109.5
C5—C4—C3	122.1 (2)	H13B—C13—H13C	109.5
C5—C4—Cl1	118.91 (18)	C8—C14—H14A	109.5
C3—C4—Cl1	119.00 (19)	C8—C14—H14B	109.5
C6—C5—C4	118.2 (2)	H14A—C14—H14B	109.5
C6—C5—H5	120.9	C8—C14—H14C	109.5
C4—C5—H5	120.9	H14A—C14—H14C	109.5
C5—C6—C1	121.0 (2)	H14B—C14—H14C	109.5
C5—C6—H6	119.5	C10—C15—H15A	109.5
C1—C6—H6	119.5	C10—C15—H15B	109.5
C12—C7—C8	120.50 (19)	H15A—C15—H15B	109.5
C12—C7—N1	118.00 (18)	C10—C15—H15C	109.5
C8—C7—N1	121.48 (17)	H15A—C15—H15C	109.5
C9—C8—C7	117.46 (19)	H15B—C15—H15C	109.5
C9—C8—C14	120.0 (2)	C7—N1—S1	120.99 (14)
C7—C8—C14	122.6 (2)	C7—N1—H1N	115.2 (16)
C8—C9—C10	123.1 (2)	S1—N1—H1N	110.7 (16)
C8—C9—H9	118.4	O1—S1—O2	118.92 (10)
C10—C9—H9	118.4	O1—S1—N1	108.16 (9)
C11—C10—C9	117.5 (2)	O2—S1—N1	105.05 (9)
C11—C10—C15	121.8 (2)	O1—S1—C1	109.29 (9)
C9—C10—C15	120.7 (2)	O2—S1—C1	107.58 (9)
C12—C11—C10	121.1 (2)	N1—S1—C1	107.26 (9)
C12—C11—H11	119.5		
			
C6—C1—C2—C3	−0.1 (3)	C8—C9—C10—C11	0.9 (3)
S1—C1—C2—C3	176.24 (15)	C8—C9—C10—C15	−179.4 (2)
C6—C1—C2—C13	179.5 (2)	C9—C10—C11—C12	−0.6 (3)
S1—C1—C2—C13	−4.2 (3)	C15—C10—C11—C12	179.7 (2)
C1—C2—C3—C4	−0.3 (3)	C10—C11—C12—C7	−0.6 (3)
C13—C2—C3—C4	−179.9 (2)	C8—C7—C12—C11	1.5 (3)
C2—C3—C4—C5	0.8 (3)	N1—C7—C12—C11	179.90 (18)
C2—C3—C4—Cl1	−179.61 (16)	C12—C7—N1—S1	77.4 (2)
C3—C4—C5—C6	−1.0 (3)	C8—C7—N1—S1	−104.2 (2)
Cl1—C4—C5—C6	179.48 (17)	C7—N1—S1—O1	−50.32 (18)
C4—C5—C6—C1	0.6 (3)	C7—N1—S1—O2	−178.29 (15)
C2—C1—C6—C5	−0.1 (3)	C7—N1—S1—C1	67.45 (17)
S1—C1—C6—C5	−176.70 (17)	C6—C1—S1—O1	−153.32 (15)
C12—C7—C8—C9	−1.2 (3)	C2—C1—S1—O1	30.2 (2)
N1—C7—C8—C9	−179.55 (18)	C6—C1—S1—O2	−22.91 (18)
C12—C7—C8—C14	178.5 (2)	C2—C1—S1—O2	160.60 (16)
N1—C7—C8—C14	0.1 (3)	C6—C1—S1—N1	89.65 (16)
C7—C8—C9—C10	0.0 (3)	C2—C1—S1—N1	−86.84 (18)
C14—C8—C9—C10	−179.7 (2)		

### Hydrogen-bond geometry (Å, °)

**Table:**

*D*—H···*A*	*D*—H	H···*A*	*D*···*A*	*D*—H···*A*
N1—H1N···O2^i^	0.83 (2)	2.27 (2)	3.072 (2)	162 (2)

Symmetry codes: (i) −*x*, −*y*+1, −*z*+2.
